# Promising Therapeutic Targets for Treatment of Rheumatoid Arthritis

**DOI:** 10.3389/fimmu.2021.686155

**Published:** 2021-07-09

**Authors:** Jie Huang, Xuekun Fu, Xinxin Chen, Zheng Li, Yuhong Huang, Chao Liang

**Affiliations:** ^1^ Department of Biology, Southern University of Science and Technology, Shenzhen, China; ^2^ Institute of Integrated Bioinfomedicine and Translational Science (IBTS), School of Chinese Medicine, Hong Kong Baptist University, Hong Kong, China

**Keywords:** rheumatoid arthritis, targets, proteins, small molecular metabolites, epigenetic regulators

## Abstract

Rheumatoid arthritis (RA) is a systemic poly-articular chronic autoimmune joint disease that mainly damages the hands and feet, which affects 0.5% to 1.0% of the population worldwide. With the sustained development of disease-modifying antirheumatic drugs (DMARDs), significant success has been achieved for preventing and relieving disease activity in RA patients. Unfortunately, some patients still show limited response to DMARDs, which puts forward new requirements for special targets and novel therapies. Understanding the pathogenetic roles of the various molecules in RA could facilitate discovery of potential therapeutic targets and approaches. In this review, both existing and emerging targets, including the proteins, small molecular metabolites, and epigenetic regulators related to RA, are discussed, with a focus on the mechanisms that result in inflammation and the development of new drugs for blocking the various modulators in RA.

## Introduction

Rheumatoid arthritis (RA) is classified as a systemic poly-articular chronic autoimmune joint disease that primarily affects hands and feet. RA is pathologically manifested as immune cell infiltration, hyperplasia of the synovial lining, pannus formation, and destruction of articular cartilage and bone (1, 2). Although the exact etiology of RA is unclear, it is certain that genetic and environmental factors have influences on RA occurrence. At present, RA affects approximately 0.5% to 1.0% of the population worldwide (3), and in particular, females are at higher risk of the disease (two to three times than males) (4). RA patients typically experience morning stiffness. If left untreated, they could appear small focal necrosis, adhesion of granulation, and fibrous tissue on the articular surface, which lead to progressive joint ankylosis, destruction, deformities, and disability (5).

To date, a large number of clinical trials have been performed by scientists and clinicians for testing different types of agents in RA treatment. Some of these agents have been approved for daily clinical practice. In the first place, nonsteroidal anti-inflammatory drugs (NSAIDs), including acetylsalicylate, naproxen, ibuprofen, and etodolac, are used to alleviate pain, swelling, and decrease inflammation. NSAIDs exert their actions by inhibiting the enzymatic activity of the cyclooxygenase (COX) involved in the synthesis of prostaglandins (PG). Inhibition of COX-2 by NSAIDs blocks PG production at sites of inflammation, whereas inhibition of COX-1 in other tissues (platelets and the gastroduodenal mucosa) leads to common adverse effects of NSAIDs, such as bleeding and gastrointestinal ulceration (6). In addition, corticosteroids, like glucocorticoids, are another kind of potent anti-inflammatory drug, which modulates gene expression by binding to glucocorticoid receptors to display anti-inflammatory and immunosuppressive effects. However, their side effects include nausea, abdominal pain, ulcers, osteoporosis, and diabetes (7).

Owing to the adverse effects of NSAIDs and corticosteroids, disease-modifying antirheumatic drugs (DMARDs), a class of immunosuppressive and immunomodulatory agents, are developed to prevent and relieve RA aggression. As the first treatment strategy, conventional synthetic (cs) DMARDs like methotrexate, hydroxychloroquine, sulfasalazine, leflunomide, chloroquine, and gold salts should be used as soon as RA is diagnosed. Notably, methotrexate is preferred for use in patients. csDMARDs are popular because of their low price and good efficacies. However, their mechanisms of action are not fully understood and multiple signal pathways could be involved. If a patient shows nonresponse for csDMARDs, biological (b) DMARDs or targeted synthetic (ts) DMARDs should be added. bDMARDs (*i.e.*, adalimumab, infliximab, certolizumab, canakinumab, tocilizumab, sarilumab, and secukinumab) are monoclonal antibodies and have special targets like tumor necrosis factor (TNF)-α, interleukin (IL)-6, IL-1β, and IL-17 (8–10). tsDMARDs also have special targets, for example, janus kinases (JAK) is the special target of tofacitinib, baricitinib, filgotinib, upadacitinib, and decernotinib (11).

Although the above mentioned DMARDs have been quite successful in mitigating RA, it is still an undeniable fact that a significant proportion of patients could experience treatment failure, including nonresponse and limited efficacy (12). To achieve the maximum therapeutic effectiveness, rheumatologists recommend using combination therapy for RA patients (13, 14). For instance, the combination of methotrexate and glucocorticoid can relieve RA in about 25% of patients within 6 months. If methotrexate plus glucocorticoid is insufficient, any bDMARDs or tsDMARDs can be recommended to add to csDMARDs, such as methotrexate plus tocilizumab, methotrexate plus rituximab, methotrexate plus tofacitinib, and so on (15). Apart from nonresponse, some DMARDs do cause adverse clinical effects, such as stomatitis, exanthema, diarrhea, anemia, pneumonia, and nephritis, further aggravating the disease condition (16–18).

With the deepening of research and exploration, many molecules are identified to exert important roles and bring novel insights to prevent RA. For example, emerging protein targets like IL-4, IL-10, IL-15, IL-17, IL-18, IL-23, interleukin-1 receptor-associated kinase (IRAK)-4 have been revealed to have a strong implication with innate and adaptive immune response in RA (19). Small molecular metabolites, including prostaglandins (PGs), lipoxins (LXs), platelet-activating factor (PAF) and leukotrienes (LTs), nitric oxide (NO), and reactive oxygen species (ROS), are also vital participants and mediators in the physiopathology of RA (20). Besides, an increasing number of studies show that epigenetic regulators play important roles in RA, like non-coding RNAs, DNA methylation, RNA methylation, and histone modifications (21). Up to now, researchers have explored and developed some new agents for RA according to these classical or emerging targets. This review searched literature published between 2005 and 2021 using keywords cytokines,” “chemokines,” “protein targets,” “small molecular metabolites,” and “epigenetics,” and summarizes recent advances in these novel targets. The review provides insights that contribute to future directions and drug discoveries for the treatment of RA.

## Protein Targets for Treatment of Rheumatoid Arthritis

Currently, many agents aiming at various protein targets have been explored and tested to ease the progression of RA, and some agents have been used in the clinic to treat RA patients **(** **Table 1** **)**. In addition to cytokine targets and chemokine targets, several important proteins participate in inflammatory cellular pathways as well, such as JAK and IRAK-4.

**Table 1:** Protein targets and their agents in rheumatoid arthritis.

Targets	Agents	Phases	References
Cytokines			
TNF	Adalimumab	Marketed	(7)
	Infliximab	Marketed	(7)
	Etanercept	Marketed	(7)
	Certolizumab	Marketed	(7)
	Golimumab	Marketed	(7)
IL-1R	Anakinra	Marketed	(7)
IL-1	Canakinumab	Marketed	(7)
	Gevokizumab	Marketed	(7)
	Rilonacept	Terminated	Clinicaltrials.gov
IL-6R	Tocilizumab	Marketed	(7)
IL-6^a^	Sarilumab	Marketed	(7)
	Clazakizumab	Marketed	(7)
	Olokizumab	Marketed	(7)
	Sirukumab	Marketed	(7)
Il-2	MEDI5117	Terminated	Clinicaltrials.gov
IL-10	Dekavil	Phase 1	(22)
IL-15	AMG-714	Phase 2	(23)
IL-18	rhIL-18BP	Phase 1	(24)
IL-17	Secukinumab	Phase 3	(25)
	Ixekizumab	Phase 2	(26)
IL-17R	Brodalumab	Terminated	Clinicaltrials.gov
IFN-γ	Fontolizumab	Terminated	Clinicaltrials.gov
**Chemokines**			
CCL2	p8A MCP-1	Animal study	(27)
	ABN912	Phase 1	(28)
CCR9	CCX8037	Animal study	(29)
CX3CL1	E6011	Phase 1	(30)
CCR1	J−113863	Animal study	(31)
	BX147	Animal study	(32)
	BAY86-5047	Phase 2	Clinicaltrials.gov
	ZK811752	Phase 2	Clinicaltrials.gov
	CCX354	Phase 2	(33)
	BMS-817399	Phase 2	Clinicaltrials.gov
CCR2	MK−0812	Phase 2	Clinicaltrials.gov
	MC−21	Animal study	(34)
	MLN1202	Phase 2a	(35)
CCR5	SCH−X82	Phase 2	(32)
	Met-RANTES	Phase 2	(36)
	AZD5672	Phase 2	(37)
	Maraviroc	Terminated	(38)
	SCH351125	Phase 1b	(39)
**Other proteins**			
TLR4	NI-0101	Phase 2	Clinicaltrials.gov
GRK2	Paroxetine	Phase 2	Clinicaltrials.gov
MEK	ARRY-162	Phase 2	Clinicaltrials.gov
MMP-9	Andecaliximab	Phase 2	(40)
CD3	Otelixizumab	Phase 1	(41)
CD80	Abatacept	Marketed	(42)
BTK	ICP-022	Phase 1	Clinicaltrials.gov
	CC-292	Phase 2	(43)
	HM71224	Phase 1	Clinicaltrials.gov
Il-23	STA 5326 mesylate	Phase 2	(44)
	Guselkumab	Terminated	Clinicaltrials.gov
GM-CSF	Otilimab	Phase 3	(45)
	Gimsilumab	Phase 1	(45)
	Namilumab	Phase 2	(45)
	Mavrilimumab	Phase 2	(45)
	Lenzilumab	Terminated	Clinicaltrials.gov
**Chemokines**			
CXCL10	MDX−1100	Phase 2	(25)
CXCL12	30D8	Animal study	(46)
CXCL13	mAb470	Animal study	(47)
CXCL16	IgG1 12-81	Animal study	(48)
CXCR1/2	Repertaxin	Animal study	(49)
	DF2162	Animal study	(50)
CXCR3	SCH546738	Animal study	(51)
	AMG487	Animal study	(52)
	JN-2	Animal study	(53)
CXCR4	Plerixafor	Animal study	(54)
	T140	Animal study	(55)
	AMD3100	Animal study	(56)
CXCR7	CCX733	Animal study	(56)
CCR7	8H3-16A12	Animal study	(57)
**Other proteins**			
JAK	Tofacitinib	Approved	(58)
	Baricitinib	Approved	(58)
	Filgotinib	Phase 3	Clinicaltrials.gov
	Upadacitinib	Approved	(58)
	Peficitinib	Phase 3	(58)
	Ruxolitinib	Phase 2	Clinicaltrials.gov
	Itacitinib	Phase 2	Clinicaltrials.gov
	Tasocitinib	Phase 2	Clinicaltrials.gov
	INCB018424	Phase 2	Clinicaltrials.gov
	VX-509	Phase 3	Clinicaltrials.gov
p38 MAPK	RO4402257	Phase 2	Clinicaltrials.gov
	PH-797804	Phase 2	Clinicaltrials.gov
	VX-702	Phase 2	Clinicaltrials.gov
	BMS-582949	Phase 2	Clinicaltrials.gov
	ARRY-371797	Phase 1	Clinicaltrials.gov
	SCIO-469	Phase 2	Clinicaltrials.gov
	SB-681323	Phase 2	Clinicaltrials.gov
IRAK-4	PF-06650833	Phase 2	(59)
	BAY1834845	Phase 1	(59)
	BAY1830839	Phase 1	(59)
	CA-4948	Phase 2	(59)
CD20	Rituximab	Phase 3	(60)
	Ocrelizumab	Terminated	Clinicaltrials.gov
	Ofatumumab	Phase 3	Clinicaltrials.gov
CD11a	Efalizumab	Phase 2	Clinicaltrials.gov
BTK	M2951	Phase 2	Clinicaltrials.gov
	GS-4059	Phase 1	Clinicaltrials.gov
CD19	MDX-1342	Phase 1	Clinicaltrials.gov

TNF, tumor necrosis factor; IL-1R, IL-1β, IL-6R, IL-6^a^, IL-2, IL-10, IL-15, IL-17, IL-17R, IL-18, IL-23, interleukin (IL)-1 receptor, -1 beta, -6 receptor, -6 antibody, -2, -10, -15, -17, -17 receptor, -18, -23, respectively; TGF-β, transforming growth factor-beta; IFN-γ, interferon-gamma; GM-CSF, granulocyte-macrophage colony stimulating factor; GM-CSFR, granulocyte-macrophage colony stimulating factor receptor; Ab, antibody; JAK, Janus kinase; IRAK-4, interleukin (IL)-1 receptor associated kinase 4; p38 MAPK, mitogen-activated protein kinases; MMP-9, matrix metalloproteinase 9; CD20, CD80, CD3, CD11a, CD19, cluster of differentiation (CD)-20, -80, -3, -11a, -19, respectively; GRK2, G protein-coupled receptor kinase 2; BMP9, bone morphogenetic protein 9; TLR4, toll like receptor 4; MEK, mitogen-activated protein kinase; BTK, Bruton’s tyrosine kinase; CXCL10, CXCL12, CXCL13, CXCL16, CXC motif ligand-10, -12, -13, -16; CXCR1/2, CXCR3, CXCR4, CXCR7, CXC motif receptor-1/2, -3, -4, -7; CCL2, CC motif ligand 2; CCR1, CCR2, CCR5, CCR7, CCR9, CC motif receptor-1, -2, -5, -7, -9; CX3CL1, CX3C ligand 1.

### Cytokine Targets

Cytokines have long been explored and studied as potential targets of RA because cytokines are directly involved in the RA process, which can be classified as pro- and anti-inflammatory cytokines based on their different functions against antigen response.

Pro-inflammatory cytokines, including TNF-α, IL-1β, IL-6, IL-7, IL-15, IL-17, IL-18, IL-23, interferon (IFN)-γ, granulocyte-macrophage colony-stimulating factor (GM-CSF) have been found to govern inflammation in RA occurrence. The level of these cytokines elevated in the synovium, synovial fluid, serum, or peripheral blood of RA patients (61–67). In addition, IL-15, IL-17, IL-23, and GM-CSF have a strong relation to rheumatoid factor (RF), anti-cyclic citrullinated peptide (CCP) seropositivity, and RA activity, which could become diagnostic biomarkers for RA (64, 67–69). IL-7 would also be taken as a diagnostic biomarker for early RA because of the different levels in the stages of RA occurrence (63).

**Figure 1:**
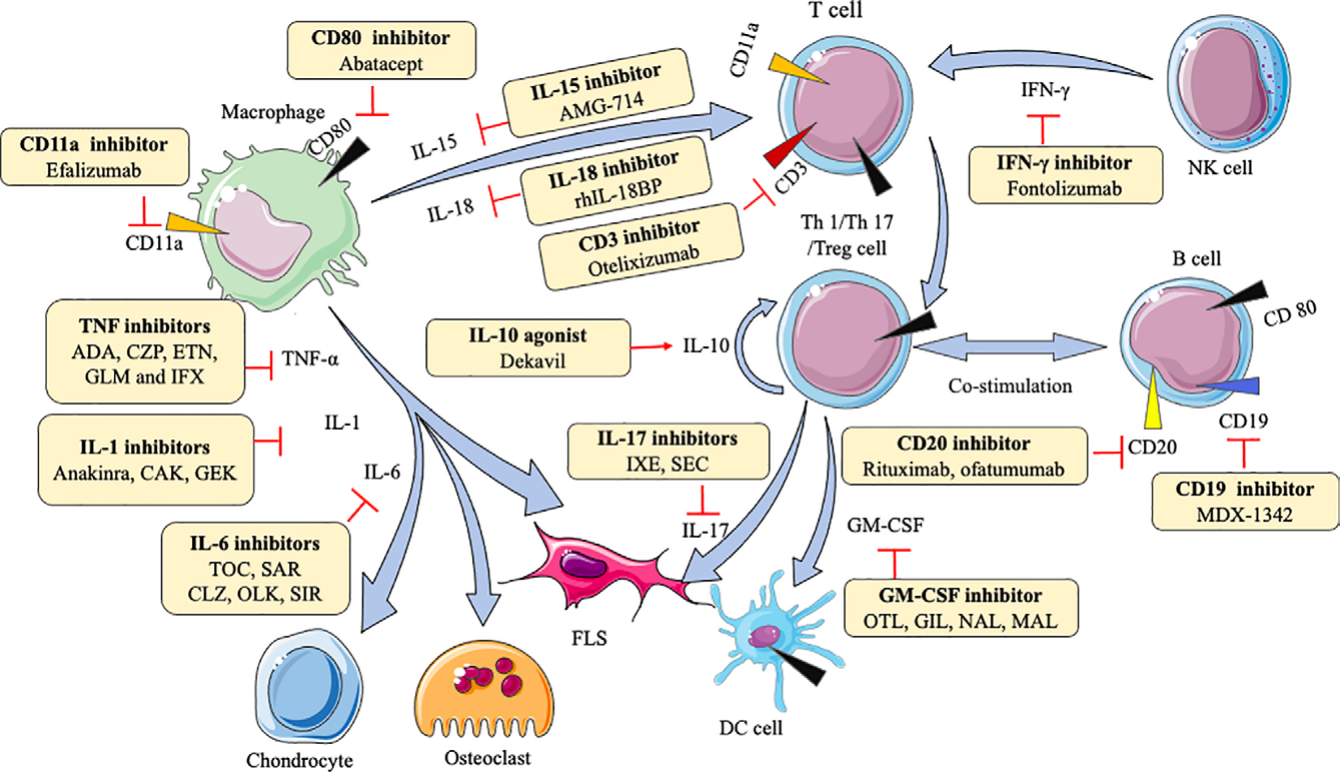
Action of drugs targeting cytokines in rheumatoid arthritis. ADA, adalimumab; CZP, certolizumab; ETN, etanercept; GLM, golimumab; IFX, infliximab; CAK, canakinumab; GEK, gevokizumab; TOC, tocilizumab; SAR, sarilumab; CLZ, clazakizumab; OLK, olokizumab; SIR, sirukumab; OTL, otelixizumab; IXE, ixekizumab; SEC, secukinumab; OTL, otilimab; GIL, gimsilumab; NAL, namilumab; MAL, mavrilimumab; FLS, fibroblast-like synoviocytes; DC, dendritic cell; NK, natural killer cell; TNF-α, tumor necrosis factor-α; IL-1, IL-6, IL-10, IL-15, IL-17, IL-18, interleukin (IL)-1, -6, -10, -15, -17, -17, respectively; IFN-γ, interferon-gamma; GM-CSF, granulocyte-macrophage colony stimulating factor.

Macrophages can secrete various cytokines, such as TNF-α, IL-1β, IL-6, IL-7, IL-15, IL-18, IL-23. TNF-α can induce the proliferation of fibroblast-like synoviocytes (FLS) and synovial cells by activating nuclear factor kappa-B (NF-κB) and extracellular regulated protein kinases (Erk)-1/2-E26 transformation-specific (ETS)-1 signaling pathway, respectively (70, 71), resulting in the secretion of a variety of inflammatory mediators like IL-6, matrix metalloproteinases (MMP)-1, and MMP-3 to increase inflammation (72). IL-1β enhances MMPs production and the adhesion of leukocytes to RA FLS by activating ERK, c-Jun N-terminal kinase (JNK), apetala (AP)-1, and NF-κB (73, 74). IL-6 causes bone resorption and cartilage degeneration by inducing the production of MMPs and NF-κB ligand (RANKL) receptors (75, 76). Blockade of IL-7 ameliorates joint inflammation by reducing T cells trafficking and proinflammatory factors like TNF-α, IL-1β, IL-6, and MMPs (77). IL-15 increases the level of major histocompatibility complex (MHC)-II on macrophages to result in enhancing proliferation of antigen-specific cluster of differentiation (CD)4^+^ T cells (78). IL-18 acts in synergy with IL-12 to stimulate T cells production of IFN-γ, which in turn stimulates synovial macrophages to produce TNF-α and IL-1β, leading to joint inflammation and cartilage destruction (79). IL-23-induced synovial inflammation is primarily linked to the activation of JAK-STAT, tyrosine kinase 2, NF-κB, and retinoic acid receptor-related orphan receptors (RORs) (80). IL-17 produced by T helper (Th) 17 cells upregulates RANKL expression, which is dependent on the IL-17/IL-17 receptor A (IL-17RA)/STAT-3 signaling cascade in FLS (81). IFN-γ is produced mainly by nature killing (NK) cells and increases CD31 and vascular cell adhesion molecule (VCAM)-1, resulting in the expansion of innate immune cell infiltration (82). Th1 cells are the predominant Th cell subset to produce GM-CSF, which can upregulate macrophage/monocyte-derived dendritic cells (Mo-DCs) numbers *via* GM-CSFR signaling (67). Understanding these mechanisms is beneficial for the development of agents in RA. As **Table 1** shows, many agents aiming at different cytokine targets have been developed and applied in practice, such as TNF inhibitors, IL-6 inhibitors, IL-1 inhibitors, IL-15 inhibitors, IL-17 inhibitors, and so on. Most of these agents act as inhibitors to affect downstream pro-inflammatory cytokines by blocking the corresponding targets, reducing symptoms and pain **(** **Figure 1** **)**. For example, adalimumab as a TNF inhibitor blocks the bind of TNF and its receptors to reduce cytokines (like MMP-1 and MMP-3)-driven inflammatory processes, which suppresses the destruction of cartilage and bone (72).

On the other hand, several cytokines, including IL-4, IL-10, IL-13, and TGF-β, exert anti-inflammatory effects in RA. Unsurprisingly, serum IL-10 level is remarkably lower in RA patients (83). A higher expression of IL-4 and IL-13 is uncovered in the synovial fluid of early RA rather than established RA, which means IL-4 and IL-13 would be the diagnostic biomarkers for early RA patients (84, 85). However, the level of TGF-β is high in the FLS and synovial fluids from RA patients (86, 87).

As anti-inflammatory factors, the injection of L-4, IL-10, IL-13, TGF-β, or their agonist can play therapeutic roles. IL-4 secreted by activated T cells has anti-angiogenic effects by inhibiting VEGF production in RA FLS, which helps relieve RA (88). IL-10, produced by regulatory T (Treg) cells, suppresses Th17 cells and promotes Treg cells in the CD4^+^ T cells population (89). IL-13 is a cytokine of Th2 cell-mediated immune response. IL-13 exerts its anti-angiogenic function *via* activation of protein kinase C (PKC) α/β II and ERK-1/2, with concomitant down-regulation of the NF-κB/p65 pathway (90), and it also can reduce the death of chondrocytes to protect the cartilage from destruction probably because of the reduction of Fc gamma receptor I (FcγRI) (91). Transforming growth factor (TGF)-β is principally expressed by macrophages and T lymphocytes. TGF-β1 promotes FLS migration and invasion by inducing epithelial-mesenchymal transition (EMT) *via* activating Smad-2/3 in RA (92). Clinical trials observed that dekavil (an agonist of IL-10) shows a significant efficacy in RA patients (93) **(** **Figure 1** **)**. These types of agents (agonist) can bind and initiate receptors to induce corresponding target reactions.

### Chemokine Targets

It is reported that chemokines are involved in the underlying pathogenesis of RA by recruiting leukocyte and affecting angiogenesis. Chemokines are divided into four categories based on different structures, which are as follows: CXC chemokines, CC chemokines, XC−chemokines, and CX3C chemokines.

CXC chemokines, including CXCL1, CXCL2, CXCL5, CXCL6, CXCL8, CXCL9, CXCL10, CXCL12, CXCL13, and CXCL16, have been identified with abnormal expression levels in synovial fluids, synovial tissues, fibroblasts, and endothelial cells of RA patients (2, 94, 95). In addition, CXC chemokine receptors also implicate in RA, such as CXCR1, CXCR2, CXCR3, CXCR4, CXCR5, CXCR6, and CXCR8. The level of these receptors is higher in RA patients than in healthy controls (96–98).

**Figure 2:**
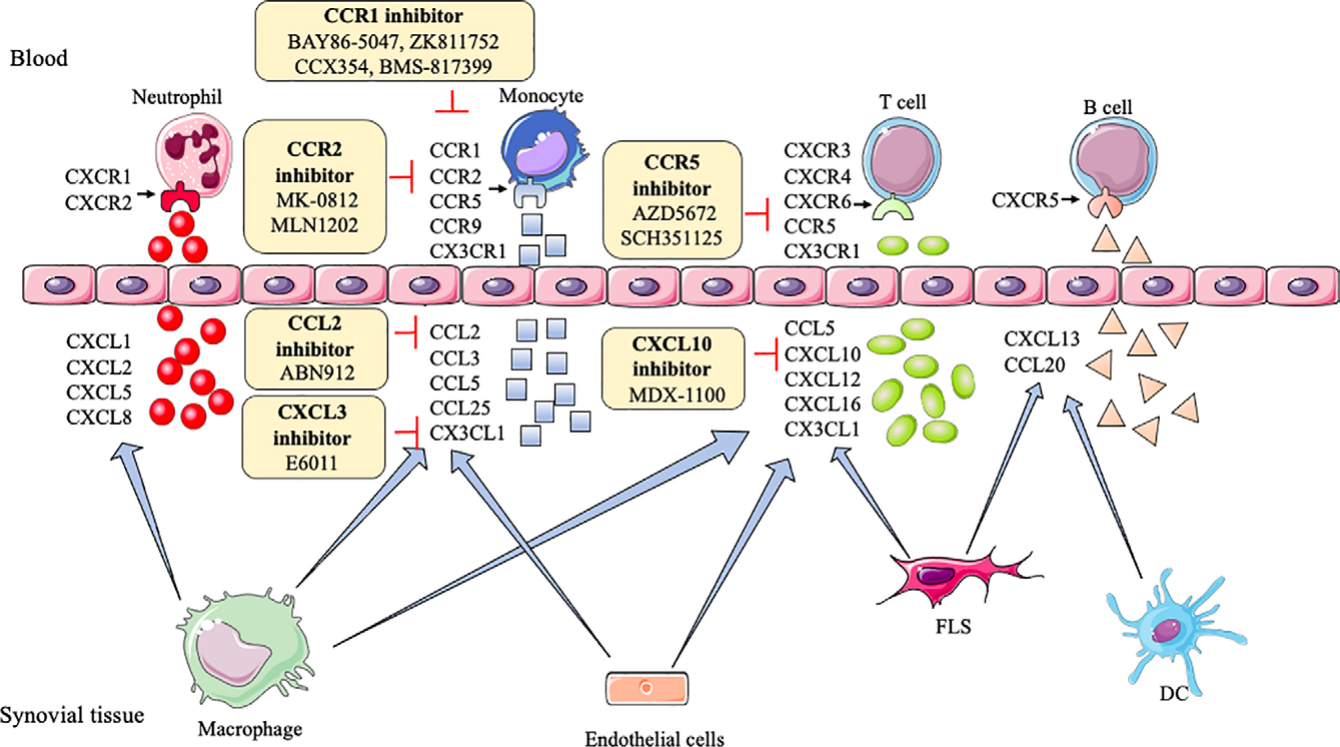
Action of drugs targeting chemokines in rheumatoid arthritis. FLS, fibroblast-like synoviocytes; DC, dendritic cell; CXCL1, CXCL2, CXCL5, CXCL8, CXCL10, CXCL12, CXCL13, CXCL16, CXC motif ligand-1, -2, -5, -8, -10, -12, -13, -16; CXCR1/2, CXCR3, CXCR4, CXCR6, CXC motif receptor-1/2, -3, -4, -6; CCL2, CCL3, CCL5, CCL20, CCL25, CC motif ligand -2, -3, -5, -20, -25; CCR1, CCR2, CCR5, CCR9, CC motif receptor-1, -2, -5, -9; CX3CL1, CX3C ligand 1.

CXC chemokines,like CXCL1, CXCL2, CXCL5, CXCL8, CXCR1, and CXCR2, generally, are involved in neutrophil chemotaxis (99), but CXCL10 and CXCL13 promote effector T cells and B cells recruitment into the joint, respectively (100, 101). CXCL12, CXCL16, and CXCR6 increase the endothelial progenitor cell recruitment and blood vessel formation in the RA joint (102). CXCR3, CXCR4, and CXCR5 enhance Th1 cells, lymphocytes, B cells, and T follicular helper (Tfh) cells into joint, respectively (100, 103). However, CXCL9 can diminish neutrophil recruitment of joints (104). As shown in **Table 1**, inhibitors or antagonists of these targets have shown good results in animals, such as CXCR3, CXCR4, CXCL10, CXCL12, and CXCL13, especially the antibody of CXCL10 (MDX-1100) has entered clinical trials (25) **(** **Figure 2** **)**.

CC chemokines including CCL2, CCL3, CCL4, CCL5, CCL7, CCL13, CCL14, CCL16, CCL18, CCL19, CCL20, CCL21, and CCL25 are abnormally expressed in plasma and synovia in RA. Levels of some CC chemokines are significantly correlated with the swollen joints, erythrocyte sedimentation rate (ESR), c-reaction protein (CRP), like CCL2, CCL5, CCL17, CCL18, CCL19 (105–107). Besides, the synovium also is rich in CCR1, CCR2, CCR3, CCR4, CCR5, CCR6, CCR7, CCR9, and CCR10 in RA (96, 108–112). CCR2, CCR4, and CCR6 are proven to positively implicate disease activity in RA (113, 114).

In RA, CCL2, CCL3, CCL4, CCL5, CCL7, CCR1, CCR2, CCR5-7, CCR9, and CCR10 induce monocytes to enter the joint synovial. CCL18, CCL19, CCL20, CCL21, CCL25, CCR5, and CCR6 recruit T cells into the joint. CCL20 induces B cells, and CCL14, CCL16, CCR3 recruit endothelial cells to enter into the inflamed joint (115–118). CCL13 has chemoattractant activity for both human myeloid leukemia mononuclear cells and human umbilical vein endothelial cells (119). CCR4 can attract skin-specific memory T cells to enter the joints (120). CCR9 can also increase the number of dendritic cells in the joint (116). Researchers find blocking or reducing these CC chemokines and their receptors, such as CCL2, CCL3, CCL5, CCL7, CCR1-5, CCR9, and CCR10, can ameliorate tissue swelling and bone erosion (27, 29, 121–124). Among them, CCL2, CCR1, CCR2, and CCR5 have achieved good clinical results (28, 33, 35, 37, 39, 125, 126) **(** **Figure 2** **)**.

XC- chemokines and their receptors (like XCL1, XCR1), CX3C chemokines and their receptors (like CX3CL1 and CX3CR1) have an up-regulation in mononuclear cells (MNCs) and FLS, respectively, in RA patients (98, 127). Many inflammatory chemokines are mainly produced by synovial macrophages and FLS in the joints of RA patients, whereas CX3CL1 is produced by synovial endothelial cells. XC and CX3C chemokines are involved in the recruitment of T cells and synovial fibroblasts. Moreover, CX3CL1 and XCL1 also promote the migration of monocytes and subchondral mesenchymal progenitor cells, respectively, into RA synovium (128, 129). Currently, a clinical trial of E6011 (an anti-CX3CL1 mAb) has been demonstrated to have a promising role in active RA patients (30) **(** **Figure 2** **)**.

### Other Protein Targets

Similar to the cytokine targets described above, many other important proteins also play remarkable roles in the pathogenesis of RA, and corresponding agents have been used in clinical settings as part of continuous research into treating RA **(** **Table 1** **)**.

In a large number of experiments, JAK, p38 mitogen-activated protein kinase (MAPK), ERK, JNK, IRAK-4, MMPs, toll-like receptor (TLR)-4, G protein-coupled receptor kinase (GRK)-2, Bruton’s tyrosine kinase (BTK), CD3, CD11a, CD19, CD20, and CD80 are demonstrated as examples of such important proteins in RA. JAK are a part of the JAK/STAT pathway, and this signaling is continuously activated, resulting in the elevated level of MMPs and apoptotic chondrocytes in RA synovial joints (130). p38 MAPK, ERK, and JNK activations are almost exclusively found in synovial. As a member of the MAPK family, p38MAPK, ERK, JNK are activated by MAPKK to influence pro-inflammatory cytokines, such as TNF, IL-6, and IL-1 (131). Specifically, p38MAPK may phosphorylate MAPKAP2, which in turn affects downstream cells (132). JNK involves in effector T cells function by stimulating Th1 differentiation in RA synovial tissue (131). IRAK-4 is an essential protein kinase in mediating pathogen recognition and local cytokine release (like IL-1, IL-6, TNF) through TLR and IL-1R signaling. Furthermore, the activity of IRAK-4 kinase regulates Th17-mediated autoimmune diseases like RA through the involvement of Th17 differentiation (133). MMPs break cartilage and bone by degrading all components of the extracellular matrix (134). TLR4 can enhance the production of pro-inflammatory cytokines and chemokines, such as IL-6 and IL-17, by binding with exogenous ligands, like peptidoglycan, in FLS and peripheral blood mononuclear (PBMC) from RA patients, and trigger cartilage inflammation and degeneration (135). GRK2 prevents the shift of M1 into M2 macrophages by mediating PGE2-EP4-cAMP-CREB signaling in synovial macrophages (136). BTK activation induces B cells survival, proliferation, and differentiation by the SYK-BTK axis (137), which is an attractive therapeutic target for RA. CD3 expressed by mature T cells and thymocytes can activate T cells signaling and regulate TCR expression by the formation of T cell receptor (TCR)/CD3 complex (138). As an adhesion molecule, CD11a can facilitate the recruitment and entry of T cells into the synovial tissue *via* LFA-1(CD11a/CD18)/intercellular cell adhesion molecule (ICAM)-1 pathway (139). CD19 amplifies the activation of Lyn and Src-family protein tyrosine kinases, thereby enhancing the signals generated by the B-cell antigen receptor to regulate B-cell development, activation, and differentiation (140). Although the biological activity of CD20 and CD80 are not fully elucidated, CD20 allows specific and effective B-cell depletion and CD80 involves in T cell co−stimulation (141). Inhibitors for these abovementioned protein targets have entered clinical trials **(** **Figure 1** **)**.

## Small Molecular Metabolite Targets for Treatment of Rheumatoid Arthritis

Previous research has shown that small molecular metabolites, like PGs, LTs, LXs, PAF, ROS, and NO, support to induce, maintain, or relieve inflammation in RA (20). Therefore, such compounds could be potential therapeutic targets (**Table 2**).

**Table 2:** Small molecular metabolite targets and their agents in rheumatoid arthritis.

Targets	Agents	Phases	References
**PGs targets**			
PGD2	MK0524	Animal study	(142)
PGE2	ER-819762	Animal study	(143)
	CR6086	Animal study	(144)
PGI2	Iloprost	Phase 2	(145)
PGJ2	15d-PGJ2	Animal study	(146)
PGF2α	AL‐8810	Animal study	(147)
TXA2	SQ29548	Animal study	(148)
**LTs targets**			
LTB4R	BIIL 284	Phase 1	(149)
CysLT1R	Montelukast	Animal study	(150)
**LXs targets**			
ALX	BML-111	Animal study	(151)
**PAF targets**			
PAFR	WB2086	Animal study	(152)
**ROS targets**			
ROS	Cinnamaldehyde	Cell culture	(153)
	Eugenol	Cell culture	(153)
**NO targets**			
iNOS	GW274150	Phase 2	(154)
	L-NAME	Animal study	(155)
**Other small molecular targets**			
CB2	HU-308	Animal study	(156)
	JWH-015	Animal study	(157)
FFAH	URB597	Animal study	(158)
	NAGly	Animal study	(159)

PGD2, prostaglandin D2; PGE2, prostaglandin E2; PGI2, prostaglandin I2; PGJ2, prostaglandin J2; TXA2, thromboxane A2; LTB4R, leukotriene B4 receptor; CysLT1R, cysteine leukotrienes 1 receptor; ALX, lipoxin A4 receptor; PAF, platelet-activating factor; PAFR, platelet-activating factor receptor; iNOS, inducible nitric oxide synthase; CB2, cannabinoid receptor 2; FFAH, specific fatty acid amide hydrolase; NAGly, N-arachidonic glycine.

### PGs Targets

**Figure 3:**
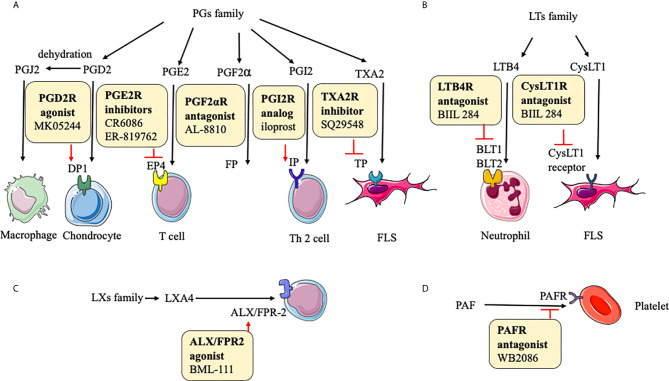
Action of drugs targeting small molecular metabolites in rheumatoid arthritis. **(A)** action of drugs targeting PGs family in rheumatoid arthritis; **(B)** action of drugs targeting LTs family in rheumatoid arthritis; **(C)** action of drugs targeting LXs family in rheumatoid arthritis; **(D)** action of drugs targeting PAF in rheumatoid arthritis. PGD2, prostaglandin D2; PGE2, prostaglandin E2; PGI2, prostaglandin I2; PGJ2, prostaglandin J2; TXA2, thromboxane A2; PGF2α, prostaglandin F2α; DP1, prostaglandin D2 receptor 1; EP4, prostaglandin E receptor 4; TP, prostaglandin TXA2 receptor; FP, prostaglandin PGF2α receptor; IP, prostaglandin PGI2 receptor; LTB4, leukotriene B4; CysLT1, cysteine leukotrienes 1; ALX, lipoxin A4 receptor; FPR-2, formyl peptide receptor-2; PAF, platelet-activating factor; PAFR, platelet-activating factor receptor; iNOS, inducible nitric oxide synthase.

In some reports, the key function of PGs is shown in physiological immune responses and pathological conditions related to inflammation and tissue damage. The expressions of PGs, including PGD2, PGE2, PGF2α, PGI2, PGJ2, and TXA2, are abnormal in RA (20). It is worth noting that PGD2 and PGJ2 are anti-inflammatory small molecules. The binding of PGD2 to DP1(a PGD receptor) inhibits IL-1–induced production of MMP-1 and MMP-13 by chondrocytes (160). It is likely that PGJ2 decreases the production of IL-1β and reactive oxygen species (ROS) through the NF-κB pathway (146). 15d-PGJ2, as the metabolite of PGD2, ameliorates disease through the suppression of Th17 cells and the induction of CD4^+^CD25^-^FOXP3 ^+^cells (146). PGE2 enhances cyclic AMP production by an EP4 (a PGE2 receptor)-dependent mechanism to increase immune inflammation (161). Although the mechanism of PGF2α in RA is unclear, it can prevent cell proliferation, inflammation, tissue remodeling *via* MMP-3, and angiogenesis *via* VEGF (147). PGI2 probably increases Th2 cell function by IP (PGI2 receptor) to reduce the production of IL-1β, IL-6, and monocyte chemoattractant protein (MCP)-1 (162). The TP receptor antagonist (SQ29548) inhibits both cyclooxygenase (COX)-2 expression and FLS proliferation, and TP agonist U46619 enhances them. Therefore, TXA2 exerts its function probably through the IP-COX-2 pathway (148) **(** **Figure 3** **)**.

### LTs Targets

In the LTs family, LTB-4 and cysteinyl (Cys)LT-1 are involved in the inflammatory response of RA. Synovial fluid LTB4 levels are upregulated in RA patients. Although the underlying mechanism of LTs is not rather clear, some trials show their function in RA. The major effect of LTB4 and its receptor is to enhance the movement of leucocytes from the circulation toward the site of tissue damage (163). An antagonist of LTB4 receptor, BIIL 284 inhibits the LTB4-stimulated expression of Mac-1 on neutrophils in RA patients (164). A potent CysLT1 receptor antagonist, montelukast inhibits the activation of the NF-κB pathway and secretion of IL-6 and IL-8 in FLS (165), next to reducing the disease incidence and its activity (166). These results mean that the inhibition of CysLT1 and LTB4 receptors would be a potential and promising new therapeutic method to prevent inflammation and disease progression in RA patients **(** **Figure 3** **)**.

### LXs Targets

The family of LXs, like LXA4 and LXB4, generated from arachidonic acid display anti-inflammatory activities. LXA4 can decrease memory B-cell response *via* engagement of lipoxin A4 receptor (ALX)/formyl peptide receptor-2 (FPR-2) in synovial tissues of patients with RA, further enhancing the reduction of inflammation (167, 168). BML-111 (an ALX/FPR2 agonist) partly downregulates the immune response in CIA (151). LXB4 has anti-inflammatory effects by regulating the adhesion and motility of monocytes and neutrophils and enhancing antibody production by human memory B cells (47, 169) (**Figure 3**).

### PAF Targets

Studies suggest that PAF plays a prominent role in RA. The activation of circulating platelets further influences leukocyte activity and participates in inflammation formation in RA patients (170). It is reported that pathways based on TNF-α regulate PAF, and TNF-α antagonists inhibit platelet activation in active patients with RA (171). WB2086, a human PAF receptor antagonist, inhibits PAF-induced platelet aggregation in animal models (152). Based on current studies, targeted agents inhibiting PAF and its receptor needed more research because of the limitation of experience with the therapeutic effects of this target.

In these mechanisms of PGs, LTs, LXs, PAF, receptors play significant roles, therefore inhibitors or agonists of these receptors can exert therapeutic functions, and the therapeutic effects are proven in animal experiments **(** **Figure 3** **)**.

### ROS and NO Targets

ROS and NO belong to oxidant molecules, which involve in the pathogenesis of many chronic autoimmune diseases, including RA. There is a strong positive correlation between serum ROS level and disease severity in both RA patients and arthritic rodent models (172). ROS regulates MAPK and NF-κB signaling pathways, further affecting cell proliferation, angiogenesis, and apoptosis in joints (173). NO and inducible nitric oxide synthase (iNOS) expressions are changed in patients with RA (174). NO regulates T and B cells infiltration by inhibiting their chemotaxis and adhesion into joints (175). Treatments with the NOS inhibitor L-NAME and iNOS inhibitor GW274150 are proven to improve inflammation response, and a trend toward a reduction in synovial thickness is observed (154, 155).

### Other Small Molecular Targets

The receptor activation of cannabinoid (CB)-1/2 involves the production of endocannabinoids, subsequently, endocannabinoids are quickly metabolized by specific fatty acid amide hydrolase (FAAH) (176). The levels of CB1 and CB2 increase in the synovial membrane with RA. CB2 inhibits IL-1β–induced proliferation of RA FLS and the activation of MAPK pathway (157). In addition, the reduction of arthritis severity and activity in FAAH knock-down mice is observed (158). Similarly, the treatment with URB597 and N-arachidonic glycine (FAAH inhibitors) prevents the occurrence of CIA in mice as well (158, 159). These results mean that the new agents targeting the endocannabinoid system are potential and promising therapeutics for further research.

## Epigenetic Targets for Treatment of Rheumatoid Arthritis

Epigenetic modifications can regulate gene expression without altering the DNA sequence. Non-coding RNAs (ncRNAs) regulation, DNA methylation, RNA methylation, and histone modifications are seen as the main mechanisms of epigenetic regulations. Numerous research has established that several abnormalities in these mechanisms eventuate in the development of RA. Corresponding target agents have been applied for patients **(** **Table 3** **).**

**Table 3:** Epigenetic targets and their agents in rheumatoid arthritis.

Targets	Agents	Phases	References
**DNA methylation**			
DNMT	Azacitidine	Animal study	(177)
	Decitabine	Animal study	(177)
	Procainamide	Animal study	(177)
	Hydralazine	Animal study	(177)
	EGCG	Animal study	(177)
**RNA methylation**			
METTL3	–	Cell culture	(178)
**Histone modification**			
HAT	Delphinidin	Cell culture	(179)
	Anacardic acid	Animal study	(180)
HMT	GSK-J4	Animal study	(181)
	EZH2	Cell culture	
HDAC	MS-275	Animal study	(182)
	Entinostat	Cell culture	(183)
**Histone modification**			
HDAC	MI192	Cell culture	(183)
	Trichostatin A	Cell culture	(183)
	Valproic acid	Animal study	(183)
	Vorinostat	Cell culture	(183)
	Nicotinamide	Cell culture	(184)
	MPT0G009	Animal study	(183)
	CKD-506	Animal study	(185)
	CKD-L	Animal study	(186)
	NK-HDAC-1	Animal study	(187)
	SAHA	Animal study	(182)
	Largazole	Cell culture	(188)
	Givinostat	Cell culture	(189)
BET	I-BET151	Animal study	(190)
	JQ1	Animal study	(191)

DNMT, DNA methyltransferase; EGCG, epigallocatechin-3-gallate; METTL3, methyltransferase-like 3; HAT, histone acetyltransferase; HDAC, histone deacetylases; HMT, histone methyltransferase; BET, bromodomain and extra-terminal; SAHA, suberoylanilide hydroxamic acid; EZH2, zeste homolog 2.

### ncRNAs

With the increasing advancement of bioinformatics analysis and microarray sequencing techniques, tremendous ncRNAs are identified in different tissues. Compared with healthy individuals, aberrant levels of abundant ncRNAs are observed, including microRNAs (miRNAs), long non-coding RNAs (lncRNAs), and circular RNAs (circRNAs). Because a large number of ncRNAs especially miRNAs are found, ncRNAs involved in the development and progression of RA are summarized in this review since 2019 sourcing from PubMed **(** **Table 4** **)**.

**Table 4:** NcRNAs targets in rheumatoid arthritis.

NcRNAs	Expression	Tissue	Signalings	Phases	References
**miRNAs**					
miR-138	Up	FLS	NF-κB signaling	Cell culture	(192)
miR-34a-3p	Down	FLS	–	Animal study	(193)
miR-23b	Up	FLS, STs	–	–	(31)
miR-125	Down	ST	PI3K/Akt/mTOR pathway	Cell culture	(194)
miR-27b-3p	Down	ST	HIPK2 signaling	Cell culture	(195)
MiR-19a-3p	Up	ST	IGFBP5 signaling	Cell culture	(196)
	Down	Plasma	SOCS3	Cell culture	(197)
miR-142-3p	Up	ST, FLS	NF-κB signaling	Cell culture	(198)
miRNA‐135a	Up	ST	PI3K/AKT pathway	Cell culture	(199)
miR-192-5p	Down	BM-MSC-exos	–	–	(200)
miR-98	Up	FLS	IL-10 signaling	Cell culture	(201)
miR-129-5p	Down	FLS	IGF-1R/SRC/ERK/EGR-1 pathway	Cell culture	(202)
miR-26a-5p	Up	FLS	PTEN/PI3K/AKT pathway	Cell culture	(203)
miR-221/222	Up	PBMC	–	–	(204)
miR-191	Up	FLS	miR-191-C/EBPβ pathway	Cell culture	(205)
miR-449a	Down	ST	HMGB1 signaling	Cell culture	(206)
miR-410-3p	Down	SF, FLS	NF-κB signaling	Cell culture	(207)
miR-506	Down	ST, FLS	TLR4 signaling	Cell culture	(208)
miR-320a	Down	ST	MAPK-ERK1/2 pathway	Cell culture	(209)
miR-29b	Up	PBM	HBP1 signaling	Cell culture	(210)
miR-155	Up	ST	FOXO3a signaling	Cell culture	(211)
miR−145−5p	Up	FLS	NF−κB pathway	Animal study	(212)
miR-22	Down	FLS	IL6R signaling/NF-κB pathway	Cell culture	(213)
	Down	ST	SIRT1 signaling	Cell culture	(214)
miRNA-141-3p	Down	FLS	FoxC1/β-catenin axis	Animal culture	(215)
miR-101-3p	Down	FLS	PTGS2 signaling	Cell culture	(216)
miR-495	Down	FLS	β-catenin pathway	Cell culture	(217)
miRNA-17-5p	Down	FLS	JAK/STAT pathway	Animal study	(218)
miRNA-140-5p	Down	FLS	STAT3 signaling	Cell culture	(219)
miR-3926	Down	FLS	TLR 5 signaling	Cell culture	(220)
miR-613	Down	FLS, ST	DKK1 signaling	Cell culture	(221)
miR-15	Up	FLS	NF-κB pathway	Cell culture	(222)
miR-21	Down	FLS	Wnt pathway	Animal study	(223)
miRNA-15a/16	Down	FLS	SOX5 axis	Cell culture	(224)
miRNA-155	Up	FLS	–	–	(225)
miR-26a	Down	CT, AC	CTGF signaling	Animal study	(226)
miR-106b	Down	SFDE	PDK4 signaling	Cell culture	(227)
miR-223	Up	FLS	–	–	(228)
miR-411	Down	ST, FLS	NF-κB pathway	Animal study	(229)
miR-9	Down	FLS	NF-κB1-RANKL pathway	Animal study	(230)
miRNA-486-5p	Down	FLS-exos	Tob1/BMP/Smad pathway	Animal study	(231)
miR-49	Up	PBMC	–	–	(232)
miR-326	Down	PBMC	–	–	(232)
miR-34a-5p	Down	ST	XBP1 signaling	Cell culture	(233)
miR-20a	Down	FLS	ADAM10 signaling	Cell culture	(234)
miR-145-5p	Down	FLS	Wnt1/β-catenin pathway	Cell culture	(235)
miR-365	Down	FLS	IGF1 signaling or PI3K/AKT/mTOR pathway	Animal study	(236)
miR-34a	Down	BM-MSC-Evs	cyclin I/ATM/ATR/p53 axis	Cell culture	(237)
miR-124a	Down	FLS	PIK3/NF-κB pathway	Cell culture	(238)
miR-9-5p	Down	Serum	REST/miR-132 pathway	Cell culture	(239)
miR-34a-5p	Down	ST	XBP1 signaling	Cell culture	(233)
**lncRNAs**					
linc01197	Down	ST	miRNA-150/THBS2 axis	Cell culture	(240)
lncRNA NEAT1	Up	PBMC- exos	miRNA-23a/MDM2/SIRT6 Axis	Cell culture	(241)
	Up	ST, FLS	miR-204-5p signaling	Cell culture	(242)
	Up	ST, FLS	MAPK/ERK pathway	Cell culture	(243)
	Up	FLS	miR-410-3p/YY1 axis	Cell culture	(244)
lncRNA PVT1	Up	FLS	miRNA-145-5p	Cell culture	(245)
	Up	ST	miR-543-dependent SCUBE2	Cell culture	(246)
	Up	FLS	SIRT6	Cell culture	(247)
lncRNA OIP5-AS1	Down	FLS	miR-448-PON1/TLR3/NF-κB axis	Cell culture	(248)
lncRNA ZFAS1	Up	FLS	miR-296-5p/MMP-15	Animal study	(249)
	Up	FLS	miR-2682-5p/ADAMTS9 axis	Cell culture	(250)
linc00152	Up	FLS	Wnt/β-catenin pathway	Cell culture	(251)
lncRNA MALAT1	Down	PBMC	Notch pathway	Cell culture	(252)
lncRNA GAS5	Down	FLS	miR-128-3p/HDAC4 axis	Cell culture	(253)
	Down	ST, FS	HIPK2 signaling	Cell culture	(254)
lncRNA HAND2-AS1	Down	MSC-exos	miR-143-3p/TNFAIP3/NF-κB pathway	Cell culture	(255)
lncRNAS56464.1	Up	FLS	miR−152−3p/Wnt pathway	Cell culture	(256)
lncRNA PICSAR	Up	FLS	miRNA-4701-5p signaling	Cell culture	(257)
lncRNA MEG3	Down	FLS	miR-141/AKT/mTOR pathway	Animal study	(258)
lncRNA ITSN1-2	Up	FLS	NOD2/RIP2 pathway	Cell culture	(259)
lncAL928768.3	Up	FLS	–	–	(260)
lncAC091493.1	Up	FLS	–	–	(260)
lncRNA HOTTIP	Up	FLS	SFRP1 demethylation	Cell culture	(261)
lncRNA HIX003209	Up	PBMC	TLR4/NF-κB pathway	Cell culture	(262)
lncRNA FER1L4	Down	ST, FLS	NLRC5 signaling	Cell culture	(263)
lncRNA CASC2	Down	Plasma	IL−17 signaling	Cell culture	(264)
lncRNA PlncRNA-1	Down	Serum, SF	TGF-β1 signaling	Cell culture	(265)
lncRNA H19	Up	FLS	Notch pathway	Cell culture	(266)
	Up	FLS	miR-124a	Animal study	(267)
lncRNA RP11-83J16.1	Down	FLS	β-catenin pathway	Cell culture	(268)
lncRNA H19	Down	FLS	NF-κB and JNK/p38 MAPK pathways	Cell culture	(269)
lncRNA XIST	Up	CT	STAT3 signaling	Animal study	(270)
lncRNA SNHG1	Up	FLS	PTBP1 signaling	Cell culture	(271)
lncRNA THRIL	Up	Serum	PI3K/AKT pathway	Cell culture	(272)
**circRNAs**					
circ_0088036	Up	FLS	miR-140-3p/SIRT 1 axis	Cell culture	(273)
circ_0000396	Down	FLS	miR-203/HBP1 axis	Cell culture	(274)
circ_AFF2	Up	FLS	miR-375/TAB2 axis	Cell culture	(275)
circ_0130438	Down	PBMC	–	–	(276)
circ_0002715	Up	PB	–	–	(277)
circ_0035197	Up	PB	–	–	(277)
circRNA_09505	Up	PBMC	miR-6089/AKT1/NF-κB axis	Animal study	(278)
circFADS2	Down	AC	miR-498/mTOR pathway	Cell culture	(279)
circ_0000175	Down	PBMC	–	–	(280)
Circ_0008410	Up	PBMC	–	–	(280)

FLS, fibroblast-like synoviocytes; CT, cartilage tissues; AC, articular chondrocytes; ST, synovial tissues; BM-MSC-Evs, bone marrow mesenchymal stem cell -derived extracellular vesicles; MSC-exos, mesenchymal stem cell-derived exosomes; PB, peripheral blood; FoxC1, forkhead box C1; PTGS2, prostaglandin-endoperoxide synthase 2; SOX5, sex determining region Y-box protein 5; CTGF, connective tissue growth factor; SFDE, synovial fibroblast-derived exosomes; PDK4, pyruvate dehydrogenase kinase 4; NF-ΚB, nuclear factor kappa-B; RANKL, receptor activator of nuclear factor-kb ligand; MDM2, murine double minute-2; SIRT6, sirtuin 6; TLR3, toll-like receptor 3; BMP, bone morphogenetic protein; Smad, mouse signal transduction molecule; MMP-15, matrix metalloproteinase 15; XBP1, X-box binding protein 1; Wnt, wingless and integration-1; ADAM, a disintegrin and metalloprotease 10; SCUBE2, signal peptide-CUB-EGF-like containing protein 2; MAPK, mitogen-activated protein kinase; ERK, extracellular regulated protein kinase; HBP1, HMG-box transcription factor 1; HDAC4, histone deacetylase 4; TNFAIP3, tumor necrosis factor alpha-inducible protein 3; TAB2, TAK1-binding 2; ATM, ataxia‐telangiectasia mutated; ATR, ATM-Rad3-related; JAK, janus kinase; STAT, signal transducers and activators of transcription; TLR 5, toll-like receptor 5; DDK1, dickkopf 1; PI3K, phosphatidylinositol 3-kinase; REST, presentational state transfer; PBM, peripheral blood monocytes; FOXO3a, forkhead box O3 alpha; SIRT6, sirtuin 6; HMGB1, high-mobility group box protein 1; C/EBPβ, CCAAT enhancer-binding proteinβ; IGF-1R, insulin-like growth factor 1 receptor; HIPK2, homeodomain-interacting protein kinase 2; SOCS 3, suppressors of cytokine signaling 3; NOD2, nucleotide-binding oligomerization domain 2; RIPK2, receptor interacting serine threonine kinase 2; NLRC5, nucleotide oligomerisation domain-like receptors 5; TGF-b1, transforming growth factor beta 1; PTBP1, polypyridine tract-binding protein 1; HIPK2, homeodomain-interacting protein kinase 2; THBS2, thrombospondin 2; PBMC, peripheral blood monouclear cell; exos: exosomes; ADAMTS9, ADAM metallopeptidase with thrombospondin type 1 motif 9; mTOR, mammalian target of rapamycinMammalian target of rapamycin.

miRNAs are small, mature, non-coding RNA molecules (about 22 nucleotides long) that can affect the processing of target mRNAs at the post-transcriptional level by translational inhibition or promoting mRNA degradation (281). Accumulating studies have revealed that altered expression and dysregulation of miRNAs have something to do with RA occurrence. miRNA-23b, miR-221/222 levels positively correlate with the ESR, rheumatoid factor (RF), CRP, disease activity score (DAS), and anti-citrullinated protein antibodies (ACPA), which will become promising targets for RA detection (31, 204). miR-16 and miR-223 are also identified as targets to distinguish patients with early RA from healthy individuals (34).

Most of the aberrant miRNA levels can alter the secretion of inflammatory cytokines or MMPs, further affecting the procession of RA. In FLS, miR-34a-3p (193), miR-129-5p (202), miR-410-3p (207), miR-506 (208), miR-22 (213), miR-101-3p (216), miR-495 (217), miRNA-17-5p (218), miRNA-140-5p (219), miR-21 (223), miRNA-15a/16 (224), miR-9 (230), miR-20a (234), miR-145-5p (235), miR-365 (236), and miR-124a (238) overexpression significantly inhibit the proliferation and promote apoptosis by aiming different proteins or other targets. On the contrary, miR-138 (192), miR-142-3p (198), miR-98 (201), miR-26a-5p (203), miR-191 (205), miR-15 (222), and miR-483-3p (282) enhance the inflammatory milieu and subsequently tissues could be damaged. Moreover, miRNA-486-5p upregulation in exosomes can repress FLS proliferation and migration, which proves exosomes to be a suitable vector for the delivery of therapeutic miRNA-486-5p (283).

As shown in **Table 4**, miRNA levels change various intracellular pathways, and the most prominent implicated pathways are those of NF-κB (192, 198, 207, 212, 213, 222, 229, 230, 238), PI3K/Akt (194, 199, 203, 236), JAK/STAT (218, 219), TLR (208, 220), β-catenin (215, 217, 235), and Wnt (223, 235). In parallel, the efficacy of several miRNAs against RA has been verified in animal experiments. Injections of miR-141-3p agomir, miR-411 mimics, miR-9, miR-21 lentivirus, miR-26a, miRNA-147 mimics have been proven to ameliorate cartilage injury and bone erosion, further inhibiting inflammatory arthritic development in CIA animal models (223, 226, 229, 230, 284). On the other hand, miRNA-17-5p lipoplex and miR−145−5p agomir given to mice are found to increase inflammatory cytokine levels (MMP−3, MMP−9, MMP−13), aggravating arthritis in the future (212, 236). The abovementioned studies about the mechanism of miRNAs have shown promising results in experimental models of arthritis, and their efficacy needs more clinical trials to prove.

Consisting of more than 200 nucleotides in length, lncRNAs are identified as long non-coding RNAs and are widely expressed in various tissues of the human body. Many studies have suggested lncRNA could become a diagnostic tool for RA. For example, lnc-AL928768.3 and lnc-AC091493.1 are positively associated with CRP, DAS, and RF (260). Apart from those two, lncRNA ENST00000483588, ENST00000456270, RNA143598, RNA143596, HIX0032090, IGHCγ1, and XLOC_002730 could also become targets to diagnose RA (285–287).

lncRNA FER1L4 and MEG3 regulate RA *via* targeting nucleotide oligomerization domain-like receptors 5 (NLRC5) in RA FLS (263, 288). Overexpression of lncRNA MEG3 plays an anti-inflammatory effect by regulating the AKT/mTOR signaling pathway (258). lncRNA PICSAR alters cell proliferation, migration, invasion, IL-6, IL-8, and MMP-3 production through sponging miR-4701-5p, in other words, lncRNA PICSAR can competitively combine miR-4701-5p to affect downstream target genes (257). As a competitive endogenous RNAs (ceRNA), lncRNA HIX003209 exaggerates inflammation through sponging miR-6089 *via* TLR-4/NF-κB pathway in RA macrophages (262). miR-222-3p/Sirt1 axis is also found to be critical for the function of lncRNA GAS5 in mitigating the proliferation, inflammation, and apoptosis of RA FLS (289). In particular, lncRNA NEAT1 and lncRNA HAND2-AS1 are found in PBMC and mesenchymal stem cells (MSC), respectively, and are involved in the regulation of RA (241, 255).

In an arthritic model experiment, silencing lncRNA ZFAS1 can mitigate inflammation and hyperplasia by competitively binding to miR-296-5p and regulating MMP-15 expression (249). Additionally, injecting lentivirus expressing shRNA for lncRNA-H19 intra-articularly at the ankle of CIA mice ameliorates the progression of CIA by competitively binding with miR-124a, which directly targets CDK2 and MCP-1 (267). The injection of lentivirus carrying sh-lncRNA XIST plasmids reduces levels of TNF-α, IL-2, and IL-6 to suppress inflammatory and damage in cartilage tissues (270). Taken together, these mechanisms described above reveal that identification of lncRNA-miRNA interaction provides new insights into the pathogenesis of RA. Thus, lncRNAs are potential and valuable targets for treating RA.

circRNAs are a category of newly endogenous non-coding RNA, and they are becoming significant members of the gene regulation environment, the most representative characteristic of which is the covalently closed RNA circle without 5′ end caps and 3′ poly tails. circRNAs have been reported to involve in the pathogenesis of some autoimmune diseases and have a wide range of functions, such as RNA polymerase (RNAP) II elongation, miRNA and RBP sponge, RNA maturation regulation, protein localization, and so on (290). Also, several circRNAs might be important targets in clinical blood samples for RA diagnosis, which show a significant association with DAS28, RF, CRP, such as circ0003972 (291), circ0002715 (277).

Several studies reveal the partial and hidden molecular mechanisms of circRNAs in the pathogenesis of RA. circ0088036 is found to be aberrantly upregulated in RA FLS, and it facilitates RA progression by acting as a miR-140-3p sponge to upregulate SIRT 1 expression (273). circFADS2 protects chondrocytes from apoptosis by acting as an interceptor in miR-498/mTOR singling pathway (292). circ_0000396 regulates miR-203/HBP1 axis to inhibit the growth of RA FLS (274). Besides, circRNA_09505 can promote AKT1 expression *via* regulating the IκBα/NF-κB signaling pathway in macrophages, the most interesting is circRNA_09505 knockdown significantly alleviates arthritis and inflammation in CIA mice (278). These emerging studies elucidate that circRNAs are potential and promising targets for RA therapy. In the future, more mechanisms of circRNAs in RA need to be uncovered.

### DNA Methylation

Methylation of DNA is carried out by the activity of DNA methyltransferases (DNMT) and leads to the formation of 5-methylcytosine (5-mC), which further affect various life activities. Altered DNA methylation patterns have been identified in clinical RA. In RA patients, an alteration of DNA methylome signature in PBMC and a reduction of 5-mC amounts in synovial tissues were observed, the alteration of FLS gene expression, including chitinase-3-like protein 1 (CHI3L1), caspase 1 (CASP1), STAT3, mitogen-activated protein kinase kinase kinase 5 (MAP3K5), familial Mediterranean fever (MEFV), and wnt1-inducible signaling protein 3 (WISP3), is caused by differentially methylated genes leading to the pathogenesis of RA (293). In addition, the change of DNA methylation is rather different between early and late stage (294, 295). Studies have shown that several DNMT inhibitors including azacitidine, decitabine, procainamide, hydralazine, and epigallocatechin-3-gallate are applied in animals, which show excellent efficacies *via* decreasing pro-inflammatory cytokines (*i.e.*, IL-6, TNF-α, and TGF) (177).

### RNA Methylation

The most common methylated modification of RNA is N6-methyladenosine (m6A). m6A methyltransferase, m6A demethylase, and m6A RNA-binding protein are essential for m6A RNA modification (296, 297). It is reported that WTAP, RIPK2, JAK3, and TNFRSF10A genes identified by transcriptome-wide high-throughput m6A sequencing are in accordance with m6A, which are increased in inflammation-related pathways, cell proliferation, and apoptosis in FLS (298). In the RA patient’s peripheral blood, m6A demethylases (ALKBH5 and FTO) and m6A RNA-binding protein (YTHDF2) are proven to have an association with DAS28, complement 3 (C3), and immunoglobulin G (IgG) (297). As a key methyltransferase of m6A, METTL3 can inhibit the activation of pTHP-1 macrophages by preventing the generation of IL-6 and TNF-α, and attenuate inflammatory response induced by LPS *via* the NF-κB signaling pathway (178). These studies provide novel prospects for us in recognizing the pathogenesis of RA and finding promising targets for RA.

### Histone Modifications

The histones can be modified in many ways posttranslationally, including acetylation, methylation, citrullination, ubiquitination, phosphorylation, and sumoylation (299, 300). These modifications implicate RA occurrence.

#### Histone Acetylation

For histone acetylation, most studies mainly focus on histone acetyltransferase (HAT) and histone deacetylases (HDAC). There are four types of HDAC, including class I (HDAC1-3, HDAC8), class II (HDAC4-7, HDAC9-10), class III (SIRT1-7), and class IV (HDAC11) (183). It has been reported that HDAC activity is involved in RA synovial are significantly increased compared with normal controls and are in direct proportion to TNF-α mRNA levels (301). In contrast, inflammatory stimuli diminish the expression of HDAC5 to regulate the generation of cytokines and chemokines through modulation of IRF1 in RA FLS (302). In addition, the mRNA and protein levels of SIRT1 in RA LFS are lower than normal FLS, and increased SIRT1 expression strikingly suppresses the invasiveness of RA FLS by inhibiting MMP1 and MMP13 expression (303). An animal study also shows that knockout specifically of SIRT1 in myeloid cells alleviates synovial inflammation and bone destruction of RA by decreasing Th1 and Th17 differentiation (304).

Two inhibitors of histone acetyltransferase (delphinidin and anacardic acid) suppress FLS proliferation and invasion, which further ameliorates inflammatory (179, 180). In addition, many HDAC inhibitors aiming at different HDAC types are also shown to suppress inflammation in cell or animal experiments, entinostat, MI192, trichostatin A, and valproic acid **(** **Table 2** **)**. These experiment results display potent therapeutic efficacies of HDAC inhibitors in RA remission (186, 305). Moreover, bromodomain and extra-terminal (BET) family proteins can identify acetylated histones. I-BET151 (a selective inhibitor of BET) can reduce joint inflammation and bone loss by blocking MMP-1, MMP-3, IL-6, and IL-8 production in RA FLS (190, 306). Similar results are seen with another BET inhibitor, JQ1, suggesting that this family of proteins may be promising therapeutic targets (191).

#### Other Histone Modifications

The involvement of histone methylation is important in the pathogenesis of RA. H3K4me3 in SF is associated with the onset of arthritis-activated chromatin. GSK-J4 can inhibit the H3K27me3 methylation at the TLR2 promoter, which significantly relieves the articular cartilage destruction and inflammation (307). The histone methyltransferase enhancer of zeste homolog 2 (EZH2) is discovered to overexpress in RA FLS and can be induced by TNF-α through the JAK and NF-κB pathways. EZH2-mediated epigenetic alteration of secreted frizzled-related protein 1 (SFRP1) significantly correlates with the activation of RA FLS (308).

## Conclusion and Perspective

RA is an autoimmune disease with complex etiology. To date, under unremitting efforts, RA has been altered from a highly disabling disease without effective treatment to a disease that can be well controlled. Many patients achieve remission or low disease state, which can be attributed to the development of specific DMARDs. However, the main problems of marketed biologics/drugs are nonresponses and partial responses as well as the occurrence of adverse effects like stomatitis, exanthema, and diarrhea. This review summarizes new targets, including proteins, small molecular metabolites, and epigenetics regulators. They are promising molecular targets for drug discovery to alleviate the onset of diseases and solve nonresponses and partial responses as well as adverse effects for current DMARDs.

It is undeniable that greater efforts are still needed to more accurately define the underlying signaling pathways affected by these newly discovered molecules and to develop appropriate therapy methods. Extensive pre-clinic studies and clinical trials also are required for proving the druggable potential of these targets. With the great success of biologic drugs targeting TNF, it seems that cytokines (*i.e.*, IL-1β, IL-6, and IL-7) and chemokines (*i.e.*, CXCL1, CCR2, and CX3CL1) could be more potent molecular targets when compared to intracellular protein targets (*i.e.*, p38MAPK, ERK, and JNK). However, some cutting-edge technologies like PROTACs (proteolysis-targeting chimeras), RNA interference, clustered regularly interspaced short palindromic repeats (CRISPR)/Cas9-based genome editing, and artificial intelligence (AI)-based drug design, allow screening of therapeutics targeting almost all kinds of molecules, like proteins and epigenetic regulators. For example, PROTACs could be used to degrade intracellular signal transducers or undruggable protein molecules. lncRNAs or miRNAs could be manipulated by small interfering RNAs, antisense RNAs, RNA mimics, or CRISPR/Cas9 carried by nanoparticle-based delivery systems. There are also inhibitors targeting methylation, acetylation, or other epigenetic modifications, which may have promising potential for RA treatment.

At present, different medication combinations are common strategies to relieve the pain and joint inflammation of RA patients. Because of the high heterogeneity of RA, researchers realize that the current efforts are far from enough to recommend specific DMARDs for individual patients. Precision medicine is an emerging medical model that considers the genetics, environment, and lifestyle of patients to select the treatment that could work best for them. Future studies could separate RA patients into subgroups and establish precision medicine strategies to realize personalized therapy.

## Author Contributions

CL designed and supervised the manuscript. JH and XF consulted literatures and wrote the manuscript in equal contribution. ZL, XC, and YH proposed advice to the manuscript. All authors contributed to the article and approved the submitted version.

## Funding

This work was supported by the Natural Science Foundation Council of China (81922081and 81700780).

## Conflict of Interest

The authors declare that the research was conducted in the absence of any commercial or financial relationships that could be construed as a potential conflict of interest.
